# Experiment and Theoretical Investigation on Fatigue Life Prediction of Fracturing Pumpheads Based on a Novel Stress-Field Intensity Approach

**DOI:** 10.3390/ma15134413

**Published:** 2022-06-22

**Authors:** Yun Zeng, Meiqiu Li, Han Wu, Ning Li, Yang Zhou

**Affiliations:** 1 School of Mechanical Engineering, Yangtze University, Jingzhou 434023, China; mechanicszy@163.com (Y.Z.); limq@yangtzeu.edu.cn (M.L.); lining@yangtzeu.edu.cn (N.L.); 2 CNOOC Ener Tech-Drilling & Production Co., Shanghai 200444, China; wuhan6@cnooc.com.cn; 3 School of Mechatronic Engineering and Automation, Shanghai University, Shanghai 200444, China

**Keywords:** fatigue life prediction, notched specimen, S-N curves, fatigue fracture region, stress-field intensity

## Abstract

Fracturing pumpheads are typical pressure vessels that experience frequent fatigue failure under the effect of notches in their cross-bore. To enhance the fatigue life of fracturing pumpheads, the study of the notch effect is indispensable and important to establish a reliable mathematical model to predict their fatigue life. In the present paper, two novel fatigue life prediction models are proposed for notched specimens. In these models, two new geometric fatigue failure regions are defined to improve the weight function. Finally, the elaborated novel stress-field intensity approach was applied to three different types of notched specimens. Experiment results indicate that the new SFI approach achieves 47.82%, 39.48%, and 31.85% higher prediction accuracy than the traditional SFI approach, respectively. It was found that the modified SFI approach provided better predictions than the traditional SFI approach and the TCD method. The II-th novel SFI approach had the highest accuracy, and the I-th novel SFI approach was more suitable for sharply notched specimens.

## 1. Introduction

In the oil and gas exploration field, with the rapid development of unconventional oil and gas drilling technology, requirements for fracturing and acidizing technology have also gradually increased. Therefore, as important components of the equipment, fracturing pumps require long life and high reliability [1]. However, the average life of pumpheads is much lower than that of other conventional components, and the manufacturing cost of pumphead components is surprisingly high, seriously affecting the economic benefits of shale gas [2,3]. Hence, it is important to accurately estimate the fatigue life of pumpheads to improve the performance of fracturing pumps.

The nominal stress method [4,5], the critical distance method [6,7,8,9], and the effective stress volume method [10,11] are most commonly used to estimate the high-cycle fatigue life of notched components. The accuracy of NSM mainly depends on the correction factor (stress concentration factor loading, stress size coefficient, roughness coefficient) and S-N curves of materials. However, owing to the complexity in petroleum engineering, empirical equations are often used to calculate the coefficients of NSM. In addition, it is proven that NSM is not suitable for predicting the fatigue life of complex structural parts [12].

**Figure 1:**
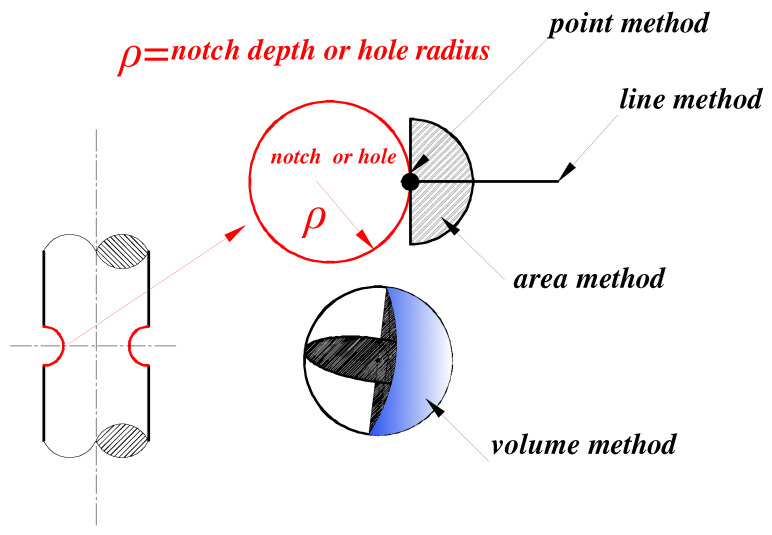
Visualization of the point method, the line method, the area method, and the volume method.

NSM considers the “hot point stress” as the core parameter of fatigue failure; however, some advanced volume methods [13,14,15] indicate that the “volume stress” in a damaged region should also be considered for fatigue failure. The critical distance method and the effective stress volume method are two main advanced volume methods. The theory of critical distance method was first proposed by Taylor and Tanaka [16,17,18,19,20,21] based on LEFM. The average stress of the maximum principal stress field in the critical region near a notch root (critical region can be a point, line, area, or volume) is regarded as a fatigue damage parameter for fatigue life estimation. The formulas for different forms of TCD can be found in [17,18,19,20]. The methods are schematically shown in Figure 1, and the formulas of the methods are as follows:

(1) $$\begin{array}{l} \mathrm{Point}\mathrm{Method}\;(\mathrm{PM}):\sigma_{mean}=\Delta \sigma_{1}\left(r=\frac{L_{0}}{2},\theta =0\right)\\ \mathrm{Line}\mathrm{Method}\;(\mathrm{LM}):\sigma_{mean}=\frac{1}{2L_{0}}\int_{0}^{2L_{0}}\sigma_{1}\left(r,\theta =0\right)dr\\ \mathrm{Area}\mathrm{Method}\;(\mathrm{AM}):\sigma_{mean}=\frac{2}{1.1\pi L^{2}{}_{0}}\int_{-\pi /2}^{\pi /2}\int_{0}^{L_{0}}\sigma_{1}\left(r,\theta \right)rdrd\theta \\ \mathrm{Volume}\mathrm{Method}\;(\mathrm{VM}):\sigma_{mean}=\frac{3}{2\pi {\left(1.54L_{0}\right)}^{3}}\int_{0}^{2\pi }\int_{0}^{\pi /2}\int_{0}^{1.54L_{0}}\sigma_{1}\left(r,\theta ,\varphi \right)r^{2}\sin\theta rdrd\theta d\varphi \end{array}$$

(2) $$El\;Haddad\;equation:\;L_{0}=\frac{1}{\pi }{\left(\frac{\Delta K_{th}}{\Delta \sigma_{-1}}\right)}^{2}$$

**Figure 2:**
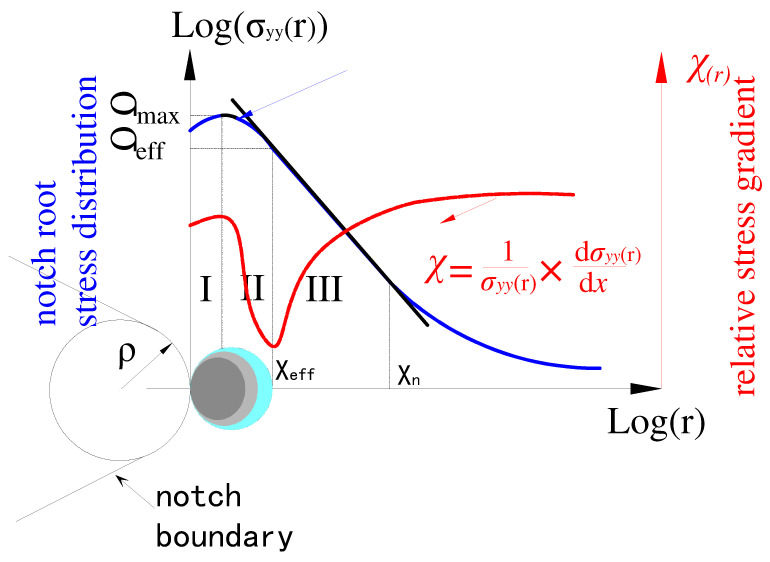
Schematic of elastic–plastic stress distribution along the notch ligament and the relative stress gradient concept.

Qylafku [22], Adib [23], and Pluvinage [24] have defined the effective distance as the length between the minimum point of the stress gradient and a notch root. Therefore, there is a certain relationship between fatigue damage and the stress gradient. The effective stress volume method introduces the concept of the effective fatigue failure region, and the calculation is used to obtain the effective stress by integrating the elastic–plastic fatigue crack-opening stress and the weight function of the effective critical damage region (schematically shown in Figure 2). The mathematical definition of the effective stress function (σ_eff_) is expressed by Equation (3):

(3) $$\begin{array}{l} \sigma_{\mathrm{eff}}=\frac{1}{X_{\mathrm{eff}}}\int_{0}^{X_{\mathrm{eff}}}\sigma_{yy}\left(x\right)\times \varphi \left(x,\chi \right)dx\\ \chi =\left|\frac{1}{\sigma_{\max}}\frac{d\sigma_{yy}\left(x\right)}{dr}\right| \end{array}$$

where *X*_eff_ is the effective distance diameter of the fatigue process zone, $\sigma_{yy}(r)$ is the fatigue crack-opening stress, $\varphi \left(r,\chi \right)$ is the relative stress gradient, and *K_ρ_* is the notch stress intensity factor.

In addition, these effective stress volume approaches have been verified in simple geometric structures; thus, it is unknown whether they are suitable for fatigue life estimation of thick-walled pressure vessels. The mean value of the fatigue failure parameter, *l*_0_ (critical distance), for aluminum alloys, cast iron, and steel is equal to 0.113 mm [25,26]. The initiation of fatigue cracks depends on grain size, grain dislocations, grain slips, and other features of microstructures. Especially, the initiation of fatigue cracks is a grain movement nucleation process at the mesoscopic scale. To simplify the calculation of the fatigue process zone, the fatigue failure parameter, *l*_0_, can be defined as the field diameter of the fatigue failure region. In addition, the plastic zone is also assumed as the fatigue damage failure region. Numerous empirical formulas are developed to calculate the cyclic plastic zone size [27,28,29,30,31,32,33] (Table 1). However, the fatigue plastic yielding zones of specimens and structures are different. In fracture mechanics, the fatigue damage failure region and the plastic yield region are defined as the fracture tip plastic zone and the crack initiation region, respectively [34,35,36,37,38]. To investigate the effects of the cyclic plastic zone on notch tip stress and fatigue life, Zhu et al. [34,35] proposed a novel approach.

Different studies have proposed different fatigue failure parameters to estimate the fatigue life of engineering components; however, these approaches are only verified on notched specimens of regular components. Fracturing pumpheads have a complex circular cross-bore structure; therefore, it is doubtful whether the fatigue failure region size can be defined as a fatigue failure parameter to predict the fatigue life of pumpheads.

In the current study, a new fatigue life prediction model was proposed for notched specimens and a novel cardioid geometric fatigue failure region was defined to improve the weight function. In addition, existing problems of the original SFI approach were corrected. The accuracy of the modified SFI approach was verified in fatigue tests on notched specimens and based on theoretical approaches for improving the fatigue life of pumpheads. 

## 2. Theoretical Analysis

### 2.1. Brief Review of Stress-Field Intensity Approach and Characteristic Parameters

#### 2.1.1. Traditional Stress-Field Intensity Approach

**Figure 3:**
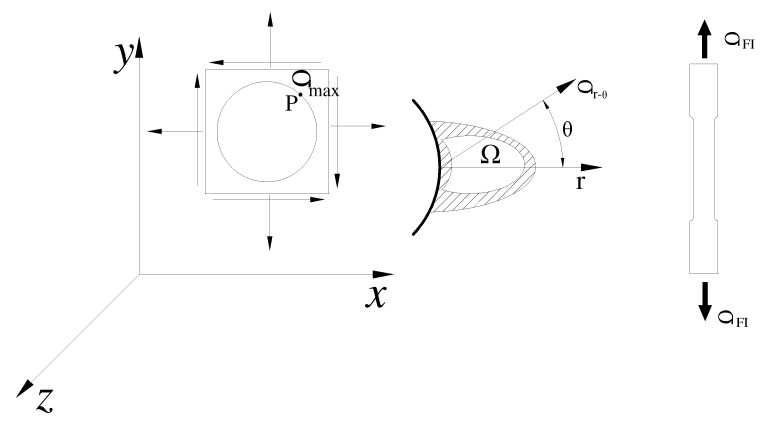
Basic model of the stress-field intensity approach.

The fatigue failure of materials occurs due to the propagation of fatigue cracks from grains. The formation of cracks can be defined as the cumulative damage of multiple grains in local areas of materials. The SFI model proposed by Yao [39] is displayed in Figure 3, and the mathematical definition of the stress-field intensity function (σ_FI_) is expressed as:

(4) $$\sigma_{FI}=\frac{1}{V}\int_{\Omega }f(\sigma_{ij})\varphi (\vec{r})dv=\frac{1}{V}\int_{\Omega }\sigma_{\left(r-\theta \right)}\varphi (r,\theta )dv$$

where $\sigma_{FI}$ is the stress-field intensity of a notched specimen, Ω is the fatigue failure region, *V* is the volume of the fatigue failure region, *R* is the field diameter, $f(\sigma_{ij})$ is the equivalent stress function, and φ(r,θ) is the weight function, which signifies the contribution of stresses at point P to the peak stress at $\left|\vec{r}\right|$.

#### 2.1.2. Parameters for Critical Fatigue State

All advanced volume methods generally include a “characteristic region parameter”. Table 2 summarizes some characteristic region parameters proposed in previous papers [6,9,10,11,17]. Although these methods were previously successfully verified, they possess some common problems.

(1) The acquisition of critical distance parameters requires the testing of numerous material constants; however, it takes a long time to experimentally obtain these material constants.

(2) The geometry of a fatigue-damaged failure area is generally defined as a common shape; hence, it is difficult to confirm whether previous advanced volume methods are suitable for calculating the fatigue damage failure region of complex pressure vessels.

(3) Owing to the size effect, the geometry and size of fatigue damage failure regions need to be reconsidered.

### 2.2. Determination of Solutions for New Fatigue Failure Regions

#### 2.2.1. Comparison of Stress Gradient Distributions near Different Notch Roots

**Figure 4:**
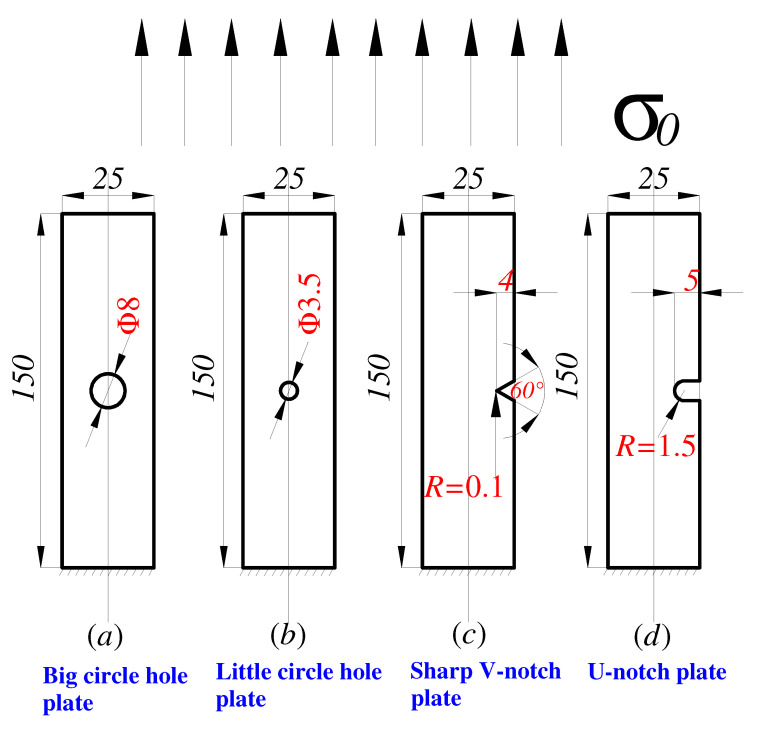
Geometries of different notched specimens (thickness = 6 mm, units: mm).

Generally, engineering structures have different and complex geometries; thus, it is difficult to select a fatigue failure region. Therefore, the determination of fatigue failure regions for notched components with common geometries is a better choice. According to the research of Susmel and Taylor [19], four different notched specimens were designed (Figure 4). Generally, FEM was applied to calculate the bisector stress distributions of the notched specimens, and LEFM and EPFM were used for calculations.

**Figure 5:**
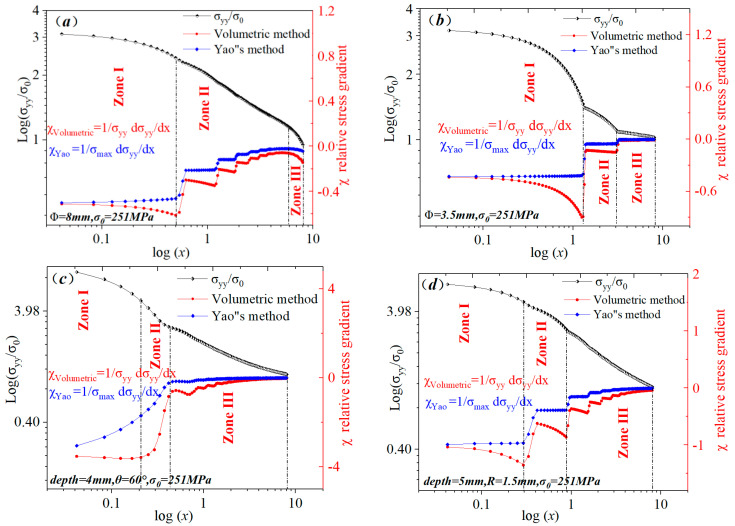
Bi-logarithmic diagrams for stress distributions at the notch bisector of different specimens at the elastic state of material. (**a**) big circle hole plate, (**b**) little circle hole plate, (**c**) sharp V-notch plate, (**d**) U-notch plate.

In the paper, finite element analysis was performed on the specimen in Figure 4, with a fixed constraint on one end and a load on the other end, and the stress distribution in the elastic state of material at the notch root bisector of each specimen was elaborated and represented by a bi-logarithmic coordinate system. Furthermore, the corresponding relative stress gradient was calculated by the volumetric method and the SFI method (Figure 5). Owing to the notch effect, relative stress gradients near the notch root of the four specimens had different forms. However, the variation trends of relative stress gradients for the four specimens were similar. According to the theory of critical distance method (TCD) [14,15,17], the minimum point of a relative stress gradient was defined as the characteristic region parameter. Figure 5 displays the elastic stress distributions and relative stress gradients near the notch root of the four specimens. Some numerical truncation errors appear based on the discrete feature of numerical calculations. Since plastic action is not considered, the relative stress gradient trend of linear elastic analysis can only be used as a reference to judge the trend. In Figure 5a,b, the trend of log(σ_0_/σ_yy_) is to be linear in zone II. On the contrary, in Figure 5c,d, the trend of log(σ_0_/σ_yy_) is to be linear in zone III. Therefore, elastoplastic analysis is also required to perform a comparative analysis of the differences between zone II and zone III.

**Figure 6:**
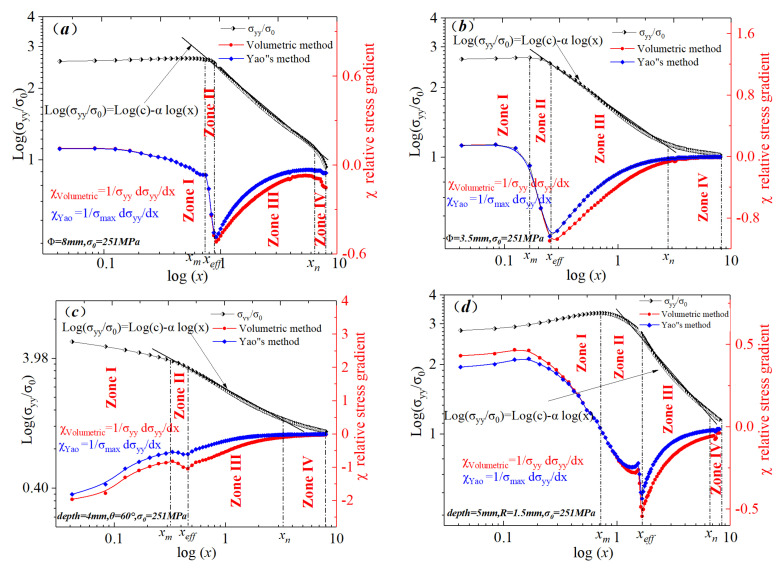
Bi-logarithmic diagrams for elastic–plastic stress distributions at the notch bisector of different specimens. (**a**) big circle hole plate, (**b**) little circle hole plate, (**c**) sharp V-notch plate, (**d**) U-notch plate.

Moreover, fatigue process zones resulted from the accumulation of the fatigue damage region, where micro- or macro-plastic cyclic strains occur. The fatigue failure region radii of the four specimens were calculated based on stress distributions for the elastic–plastic state at the notch root bisector and relative stress gradients in a bi-logarithmic coordinate system. Figure 6 displays the elastic–plastic stress distributions and relative stress gradients near the notch root of the four specimens. The peak in stress for the elastic state always appeared at the notch root surface (the origin of the *X*-axis in the Cartesian coordinate system). However, the stress peak in the elastic stress appeared at a certain distance from the notch root. The point of the stress peak and its counterpart distance, *X_m_*, were defined as zone I, which was a completely plastic region in the bi-logarithmic coordinate system. In zone II, the peak stress manifested a decreasing trend. The distribution trends of relative stress gradients in zones II and III were opposite, especially the relative stress gradient, which dropped gradually in zone II. The point at which the relative stress gradient started to manifest an increasing trend was considered as the effective distance. In zone III, the elastic–plastic stress distribution was linear; thus, a gradual plastic to elastic material transition occurred in this region. In zone IV, the elastic–plastic stress distribution was no longer linear, and the relative stress gradient expressed a new decreasing trend. The relative stress gradient was far from the notch root and had a minimum effect on fatigue failure.

#### 2.2.2. Summary of Fatigue Damage Failure Area Shapes for Different Notched Specimens

It is propounded that in addition to the “stress peak point”, the stress field in a specific region should also be considered during fatigue life analysis [6,7,36,43,44,45,46]. TCD, the volumetric method, and the SFI approach are defined as macro-mechanical methods; thus, failure criteria of these methods should be combined with the critical damage region. In this work, the SFI approach was used to predict the fatigue life of fracturing pumpheads. The shape and size of the fatigue damage region are key factors for accurate fatigue life prediction.

**Figure 7:**
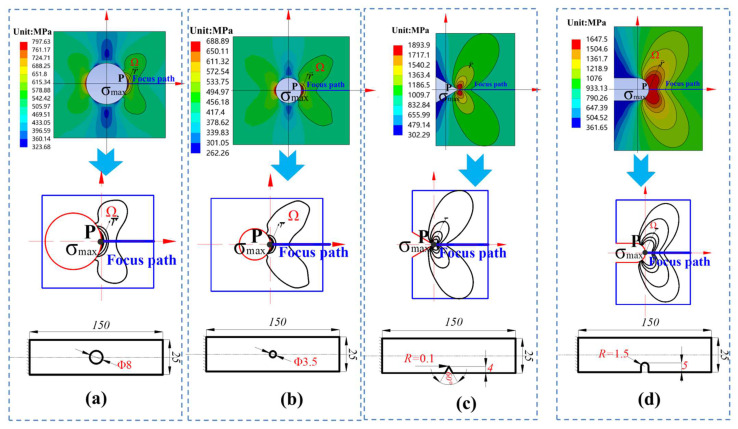
Elastic–plastic stress nephograms at the notch root of the four specimens under pressure of 251 MPa. (**a**) big circle hole plate, (**b**) little circle hole plate, (**c**) sharp V-notch plate, (**d**) U-notch plate.

Figure 7 displays the stress nephograms for the elastic–plastic stress at the notch root of the four specimens under a pressure of 251 MPa. The high-stress contours of the circular plate specimen had a crescent shape, whereas those of the V-notched and U-notched specimens had a heart shape. Obviously, regions enclosed by these high-stress contour lines were plastic. Elastic–plastic transition zones near high-stress plastic zones also had an important influence on fatigue failure. It was found that elastic–plastic stress contour lines of the four notched specimens manifested roughly similar regions with a heart shape. Therefore, it is feasible to assume that the shape of the fatigue failure region was cardioid.

### 2.3. Improvement of the Stress-Field Intensity Approach

#### 2.3.1. Traditional Stress-Field Intensity Approach

The traditional SFI approach was modified to effectively predict the fatigue life of notched components. However, the traditional SFI approach and other critical damage region approaches have several common problems.

1. Owing to the randomness of grain damage in fatigue damage failure regions and the influence of stress gradients near notch roots, uniform shapes cannot be used to define damage regions in notched components.

2. Micro-plastic cyclic strains are one of the main reasons for fatigue failure. However, macro-mechanical methods do not consider the influence of microcosmic factors. Hence, material parameters at the microstructural scale are essential factors for fatigue failure.

3. The mechanical resistance of microstructure barriers inhibits the propagation of short cracks. However, whether grains near the peak stress point at a notch root play a “contribution” or “hindrance” role in the initiation and propagation of cracks has not been clearly defined. 

#### 2.3.2. Optimization of the Traditional Stress-Field Intensity Approach

The accuracy of the traditional SFI approach depends on the failure equivalent stress function, the fatigue failure region, and the weight function. In Section 2.3.1, it is asserted that the effect of inner grains existing far away from a high-stress region also needs to be considered for fatigue life prediction. Therefore, the revised formula can be expressed as:

(5) $$\sigma_{FI}=\frac{1}{V}\int_{\Omega }f(\sigma_{ij})\varphi (\vec{r})dv=\sigma_{\max}-\xi (\Omega )$$

where ξ(Ω) is the “auxiliary part”, which can be expressed as:

(6) $$\xi (\Omega )=\frac{\int_{\Omega }(\sigma_{\max}-\sigma_{r-\theta })\varphi (r,\theta )dv}{\int_{\Omega }\varphi (r,\theta )dv}$$

**Figure 8:**
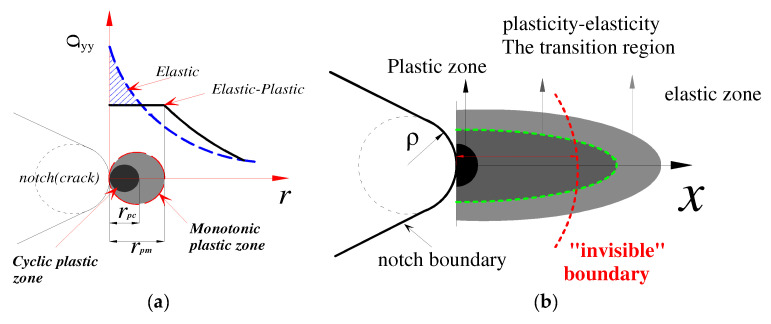
Schematic diagram of the novel elastic–plastic zone at the crack tip (notch): (**a**) monotonic plastic zone and cyclic plastic zone (**b**) invisible boundary region.

The novel method proposed in this work is different from the traditional SFI approach and does not require an artificial field diameter. It was considered that the continuous accumulation of damages resulted from the combined effect of the peak stress point and other points in the damage region. The hypothetical concept of an “invisible boundary fatigue failure region” is illustrated in Figure 8. In Figure 8a, r_pc_ is the size of the cyclic plastic zone, and r_pm_ is the size of the monotonic plastic zone.

The weight function of the traditional SFI approach is expressed as:

(7) $$\varphi (r,\theta )=1-\chi r(1+\sin\theta )=1-\left|\frac{1}{\sigma_{\max}}\frac{d\sigma_{r-\theta }}{dr}\right|r(1+\sin\theta )$$

The weight function of the traditional SFI approach is a generalized monotonically decreasing function about $\left|\vec{r}\right|$. However, it is also reported that as the distance from a notch root increases, the variation trend of the weight function does not comply with the original definition [36]. Therefore, it is important to correct the weight function for accurate fatigue life prediction. It can be inferred from Equation (9) that the weight function is mainly composed of three parts: relative stress gradient, distance function of the notch root, and function of angle *θ*.

In this work, common problems of the weight function of the traditional SFI approach, similar to those mentioned in Section 2.3.1, are summarized and revised.

(1) The direction angle of the relative stress gradient is not clearly expressed by the traditional weight function. Hence, the variable of distance $\vec{r}$ for a notch root was revised to a distance function r(θ). 

(2) Grain size is an essential factor for fatigue life prediction; therefore, it was introduced as a coefficient in the calculation formula of the weight function of the SFI approach.

(3) The weight function defines the contribution of stresses at point P to the peak stress at $\vec{\left|r\right|}$; however, considering the inhibition effect of the “auxiliary part”, (1+sinθ) is no longer applicable. Hence, the function f(θ) of angle *θ* was proposed to revise the weight function.

(4) Many researchers [43,47,48] have also proven that the grain size is related to value of the stress-intensity field; moreover, the grain size means the scale of the grain dimension. Therefore, the authors consider the grain size of the material to modify the weight function. 

Considering the above factors and the function of ξ(Ω) as a separate part, the revised formula can be expressed as:

(8) $$\varphi \left(x,\theta \right)={\left[\left|\frac{1}{\sigma_{\max}}\frac{d\sigma_{r-\theta }}{dr}\right|r(\theta )f(\theta )\right]}^{\frac{r(\theta )}{G}}$$

where *G* is the grain size and *r(θ)* is the geometric relationship function of stress contour distribution in a damaged region.

### 2.4. Hypothesis of the “Cardioid” Fatigue Damage Failure Region

Finite element analysis results revealed that fatigue failure regions of the notched specimens had two geometrical shapes. In Figure 7, the highly stressed damage volume geometry of the plate specimen with holes has a crescent shape, whereas those of the U-notched and V-notched specimens have a heart shape.

#### 2.4.1. Crescent-Shaped Fatigue Failure Region

**Figure 9:**
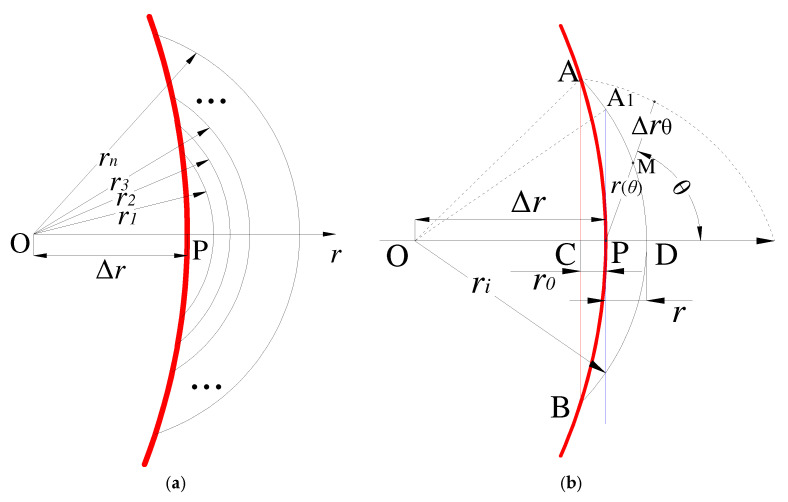
Schematic representation of the notched specimen with holes. (**a**) Stress contour lines at the notch root. (**b**) Relationship between stress contours and the peak point.

The relationship between stress contours and the peak point is geometrically expressed in Figure 9. To simplify the calculation, the approximate geometric relationships of some parameters are displayed in Figure 9b, and the corresponding differential equations are expressed as:

(9) $$\left\{\begin{array}{l} OA=OA_{1}=OD=r_{i}\\ OD=OP+PD=r_{i}=\Delta r+r\\ A_{1}P=\sqrt{OA_{1}{}^{2}-OP^{2}}=\sqrt{r_{i}{}^{2}-\Delta r^{2}}=\sqrt{r^{2}+2r\Delta r}\\ AC=\sqrt{r_{i}{}^{2}-{\left(\Delta r-r_{0}\right)}^{2}}\\ AP=\sqrt{r_{i}{}^{2}-{\left(\Delta r-r_{0}\right)}^{2}+r_{0}{}^{2}}=\sqrt{r^{2}+2\Delta r(r+r_{0})} \end{array}\right.$$

To solve these differential equations, the results of *r(θ)* were considered infinite. Therefore, in Figure 9b, lines *A*_1_*P* and *PD* are two special boundary solutions, which can be expressed as:

(10) $$\left\{\begin{array}{l} r(\frac{\pi }{2})=\sqrt{r_{i}{}^{2}-\Delta r^{2}},r(0)=r\\ {\left.r^{\prime }(\theta )\right|}_{\theta =0}=0 \end{array}\right.$$

Hence,

(11) $$r(\theta )=(2\sin^{2}\frac{\theta }{2})(\sqrt{r^{2}+2r\Delta r}-r)+r$$

#### 2.4.2. Cardioid-Shaped Fatigue Failure Region

**Figure 10:**
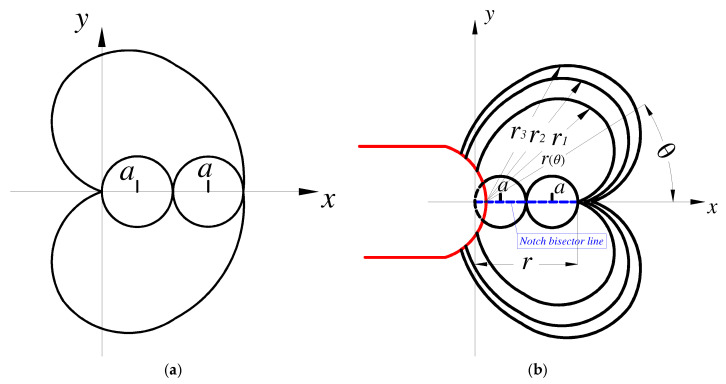
Schematic representation of the U-notched specimen. (**a**) Cartesian cardioid. (**b**) Relationship between stress contours and the peak point.

According to FEA results, the geometry volume of the fatigue failure region of the U-notched and V-notched specimens in the elastic–plastic stress had a cardioid shape. The relationship between stress contours and the peak point was expressed by periodic distribution (Figure 10).

(12) $$\left\{\begin{array}{l} x^{2}+y^{2}+ax=a\sqrt{x^{2}+y^{2}}\\ {\left(r(\theta )\right)}^{2}+ar(\theta )\cos\theta =ar(\theta )\\ r(0)=r \end{array}\right.$$

In addition, according to the concept of the “invisible boundary damage region”, the field diameter of an invisible boundary does not require an artificial definition. As the distance increased, the “inhibition” gradient (dΩ/dr) reached zero; thus, the distance was determined as the field diameter. Therefore, the field diameter of the cardioid fatigue failure region can be redefined as:

(13) $$\left\{\begin{array}{l} 2a=r\\ r(\theta )=a\left(1-\cos\theta \right) \end{array}\right.$$

#### 2.4.3. Simplification of the Advanced SFI Approach

In the traditional SFI approach, the function of angle *θ* is expressed as (1 + sin*θ*). In addition, the contributions of different stress contours to fatigue failure require different field diameters; however, none of these contributions exceed the corresponding stress values. Therefore, the function of angle *θ* can be redefined as:

(14) f(θ)=cosθ

In addition, based on Equations (8), (11)–(14), the field-intensity formula can be expressed as:

(15) $$\sigma_{FI}=\sigma_{\max}-\frac{\int_{\Omega }(\sigma_{\max}-\sigma_{r-\theta }){\left[\left|\frac{1}{\sigma_{\max}}\frac{d\sigma_{r-\theta }}{dr}\right|r(\theta )\cos(\theta )\right]}^{\frac{r(\theta )}{G}}dv}{\int_{\Omega }{\left[\left|\frac{1}{\sigma_{\max}}\frac{d\sigma_{r-\theta }}{dr}\right|r(\theta )\cos(\theta )\right]}^{\frac{r(\theta )}{G}}dv}$$

(16) $$\left\{\begin{array}{l} I-crescent:r(\theta )=(2\sin^{2}\frac{\theta }{2})(\sqrt{r^{2}+2r\Delta r}-r)+r\\ II-cardioid:r(\theta )=\frac{r}{2}\left(1-\cos\theta \right) \end{array}\right.$$

Considering the symmetry of a notched specimen, the simplified field-intensity calculation formula can be expressed as:

(17) $$\sigma_{FI}=\sigma_{\max}-\frac{\int_{0}^{\infty }\int_{0}^{\frac{\pi }{2}}(\sigma_{\max}-\sigma_{r}){\left[\left|\frac{1}{\sigma_{\max}}\frac{d\sigma_{r}}{dr}\right|r(\theta )\cos(\theta )\right]}^{\frac{r(\theta )}{G}}d\theta dr}{\int_{0}^{\infty }\int_{0}^{\frac{\pi }{2}}{\left[\left|\frac{1}{\sigma_{\max}}\frac{d\sigma_{r}}{dr}\right|r(\theta )\cos(\theta )\right]}^{\frac{r(\theta )}{G}}d\theta dr}$$

## 3. Materials and Experiment

### 3.1. Specimen Geometry

**Figure 11:**
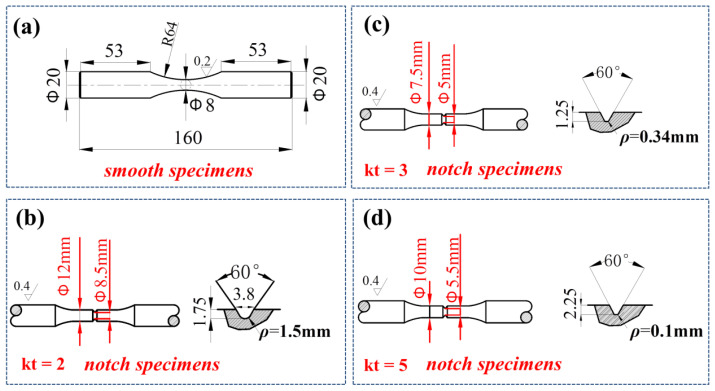
Geometries and dimensions of test specimens (in mm) used for S-N curve and notch specimens tests: (**a**) smooth specimen, and (**b**) circumferentially notched solid-bar specimen with kt = 2, (**c**) kt = 3, and (**d**) kt = 5.

**Figure 12:**
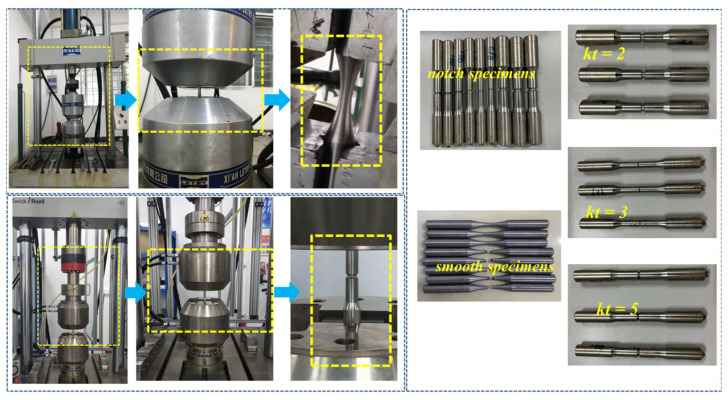
Illustration of the specimen clamping mechanical testing system.

The geometry and dimensions of the smooth solid-bar specimen used for the S-N curve test are presented in Figure 11a. In addition, three notched specimens of different elastic SCFs (kt) were designed to verify the reliability of the new approach, and their geometries and dimensions are presented in Figure 11b–d. All fatigue experiments were conducted on test equipment (Figure 12).

### 3.2. S-N Curve Test Procedure

#### 3.2.1. Static Mechanical Properties of Test Material

According to the GB/T 3075-2008 standard and ISO 6892:1998, the mechanical properties of the test material (40CrNi_2_MoVA) were tested prior to the S-N curve experiment (Table 3).

#### 3.2.2. Uniaxial Test

Uniaxial fatigue tests were conducted on a PLD-300 electro-hydraulic servo fatigue testing machine (Figure 12), and an axial tensile sinusoidal waveform load was selected to control the process. The stress ratio (*R* = −1) was constant under the varying frequencies of 10–30 Hz. Specimens’ buckling was reduced to a minimum because the shapes of measurement distances were represented by hourglass sections and notches that become the weakest regions. It means fatigue damages have occurred in the mentioned zones, while avoiding the other ones (Figure 12). According to the value of ultimate tensile strength (*σ*_b_), 35 specimens were divided into 7 groups with different stress levels. According to the GB/T 24176-2009 standard and ISO 12107:2003, the confidence level of the S-N curve of 40CrNi_2_MoV was obtained as 50%. *N_f_* is the fatigue life recorded for each experiment. S-N curve fitting was carried out by using the two-parameter method, and the fitting formula can be expressed as:

(18) $$\sigma^{m}\times N=C$$

where *m* and *C* are fitted values, *σ* is the test stress, and *N* is the number of cycles to fracture.

**Figure 13:**
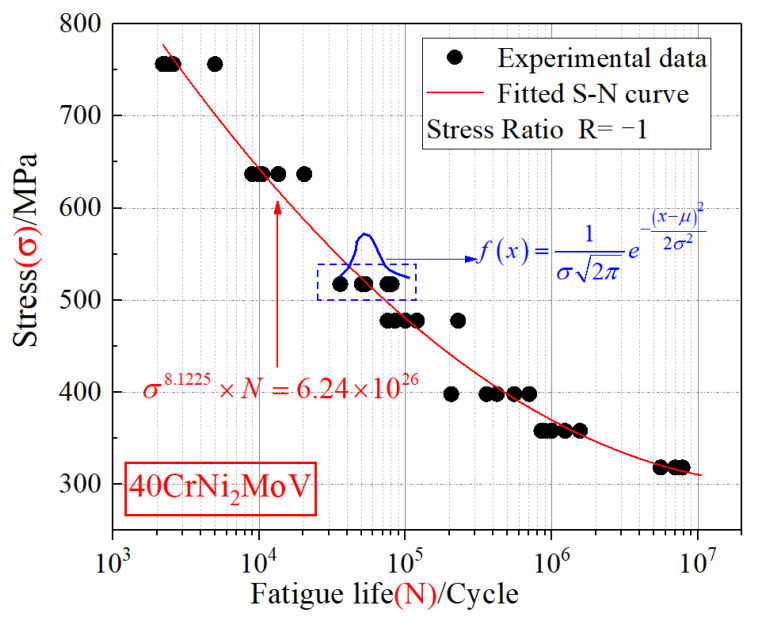
Fatigue data and fitted S-N curve for the pumphead material.

Results of the S-N curve experiment are presented in Table 4, and the fitted S-N curve is plotted in Figure 13.

### 3.3. S-N Curve Test of Notched Specimens

S-N curve tests of three different notched specimens were conducted on a Zwick HB250 fatigue testing machine Figure 12. Based on the actual working condition of the pumphead, axial tensile sinusoidal waveform loading with R = −1 was selected to control the testing machine. The loading conditions and fatigue experimental results of the notched specimens are presented in Table 5.

## 4. Results and Discussion

### 4.1. Fatigue Life Verification of Notched Specimens

**Figure 14:**
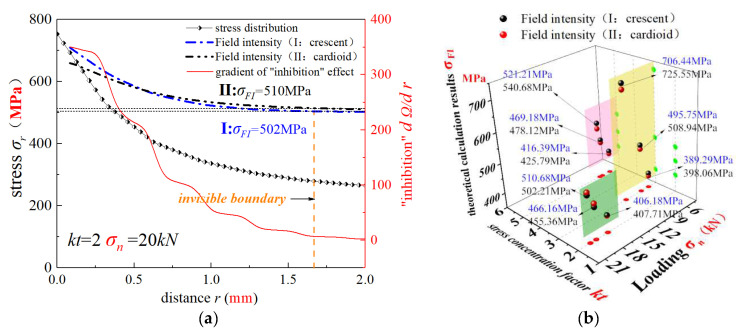
Analysis of stress fields near the notch by the simplified and improved SFI approach: (**a**) loading result for kt = 2 and $\sigma_{n}=20kN$, and (**b**) theoretical calculation results for 9 different cases.

In Section 2, the theoretical SFI approach and the numerical simulation method were introduced. According to experimental loading conditions, three different stress levels were set for each notched specimen prior to the elastic–plastic finite element analysis. The static structural module of ANSYS workbench software was used for numerical calculations. To ensure the accuracy of FEA results, meshes at the notch root of the specimens were refined. In Section 2.4, two different geometrical shapes of the fatigue failure region (crescent and cardioid) were proposed; thus, the improved SFI approach was also divided into two models (Equations (12) and (13)). Extracting stress distribution data along the symmetry line (focus path) of the notched specimens, stress distributions and stress-field intensities were calculated by the novel SFI approach (Figure 14). The stress gradient decreased rapidly in highly stressed volumes, and the “inhibition” gradient presented a similar trend (Figure 14a). However, with the increase of the field diameter, the “inhibition” gradient gradually approached zero and the stress-field intensity gradually changed to a constant value. The same trend was noticed for the other loading cases, and the corresponding results are presented in Figure 14b and Table 6.

**Figure 15:**
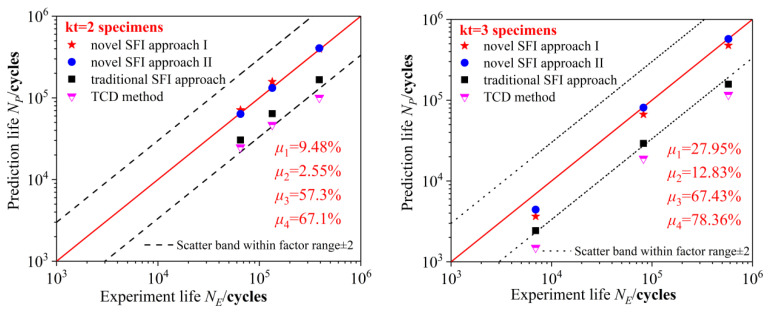

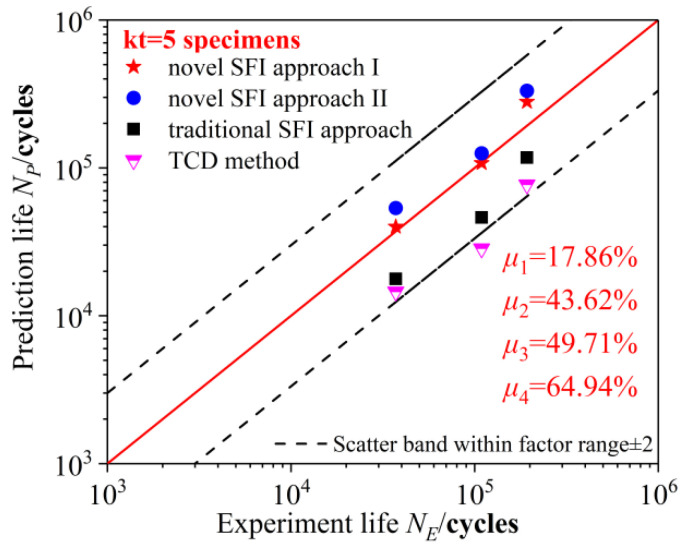
Index parameters of notched specimens calculated by four different approaches.

Based on the aforementioned principle, two other approaches (traditional SFI approach and TCD method) were chosen to calculate the fatigue life of the notched specimens. In comparison to the traditional SFI approach and the TCD method, the novel SFI approach had the narrowest error band (Figure 14), indicating its higher fatigue life prediction accuracy. The error mean absolute percent index parameter (*μ*) was expressed as:

(19) $$\mu =\frac{1}{n}\sum_{i=1}^{n}\left(\left|\frac{N_{P}-N_{E}}{N_{E}}\right|\times 100\%\right)$$

where *N_P_* is the predicted life and *N_E_* is the experimental life. The index parameters of the notched specimens are presented in Figure 15.

The fatigue life prediction accuracies of different approaches were quantitatively analyzed. The novel SFI approach II had the highest fatigue life prediction accuracy for the kt = 2 notched specimen. For the kt = 2 notched specimen, the deviations between the novel SFI approach II-based calculation results and the experimental fatigue life values under 20, 19, and 17 kN were 2.59%, 1.35%, and 3.72%, respectively. For the kt = 3 notched specimen, the deviations between the novel SFI approach II-based calculation results and the experimental fatigue life values under 10, 7, and 5.5 kN were 36.2%, 1.61%, and 0.59%, respectively. For the kt = 5 notched specimen, the deviations between the novel SFI approach II-based calculation results and the experimental fatigue life values under 8, 7.5, and 6 kN were 43.5%, 14.9%, and 72.3%, respectively. Similarly, for the kt = 2 notched specimen, the deviations between the novel SFI approach II-based calculation results and the experimental fatigue life values under 20, 19, and 17 kN were 8.9%, 17.6%, and 1.79%, respectively. For the kt = 3 notched specimen, the deviations between the novel SFI approach II-based calculation results and the experimental fatigue life values under 10, 7, and 5.5 kN were 47.4%, 18.9%, and 17.3%, respectively. For the kt = 5 notched specimen, the deviations between the novel SFI approach II-based calculation results and the experimental fatigue life values under 8, 7.5, and 6 kN were 7.3%, 1.43%, and 44.8%, respectively.

Therefore, when the stress concentration factors were 2 and 3, the SFI approach II was more accurate in predicting fatigue life; however, when the stress concentration factor was 5, the SFI approach I had better fatigue life prediction efficiency than the SFI approach II. In terms of the absolute percent index parameter (*μ*), the SFI approach I (25.76%) was more accurate than the SFI approach II; thus, the SFI approach I was more suitable for the fatigue life prediction of sharp notched specimens.

The deviations between fatigue life values calculated by the traditional SFI approach and the TCD method under different loading conditions were approximately 70%. Therefore, both the novel SFI approaches I and II are highly accurate in predicting high-cycle fatigue life.

### 4.2. Fatigue Life Verification of Pumphead

**Figure 16:**
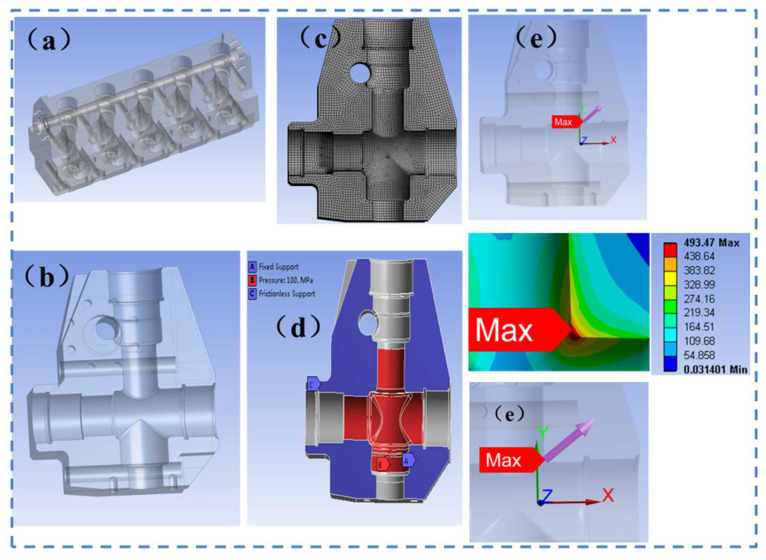
(**a**) Analysis model of the hydraulic part of the fracturing pump, (**b**) 1/2 inner cavity of the pumphead, (**c**) typical mesh structure, (**d**) boundary conditions for numerical simulations, and (**e**) maximum stress contour and focus path.

To further verify the fatigue life prediction accuracy of the novel SFI approach for the pumphead, numerical simulations for stress distribution and fatigue life were performed in ANSYS workbench software (Figure 16).

**Figure 17:**
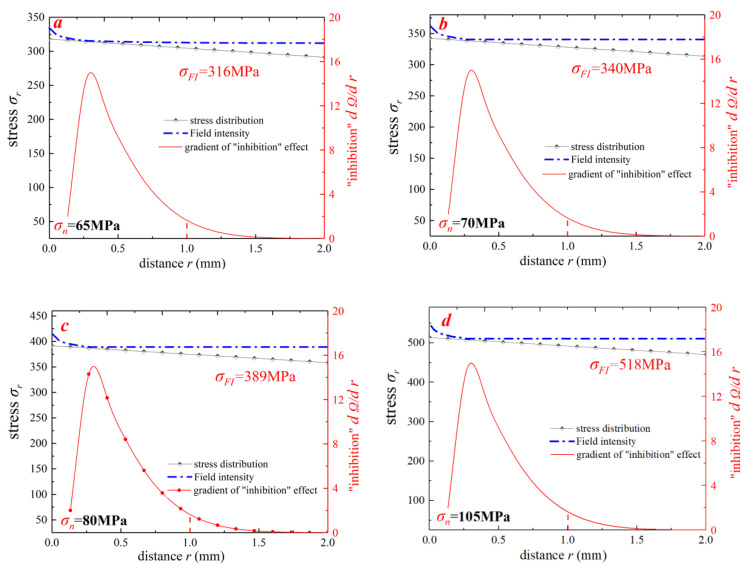
Simplified novel SFI method to analyze the stress field near the concentration point of the intersecting line under the four load conditions: (**a**) 65 MPa, (**b**) 70 MPa, (**c**) 80 MPa, and (**d**) 105 MPa.

For extracting stress distribution data along the symmetry line (focus path) of the pumphead cross-bore, field intensities under four different working conditions were calculated by the novel SFI approach II. The gradient value of “inhibition action” was calculated as shown in Figure 17. As shown in Figure 17, the slope of the “inhibition” effect (dΩ/dr) is close to 0 in almost all the four working conditions when R is close to 1.5 mm. With the increase of the field diameter, the value of field strength does not alter and steadily becomes constant. It can be judged that the damage in this area can be defined as the effective damage area of the ultra-high-pressure pumphead body. The field intensity under 65, 70, 80, and 105 MPa was 318, 340, 389, and 518 MPa, respectively. Further, substituting these four field strengths into the S-N curve of the initial pumphead material, the fatigue life was calculated by the new method.

According to the actual working conditions of the fracturing pump, the loading frequency of the circular cross-bore was 110 cycles/min. Owing to the component size, material differences, and loading distribution, even under the same operating conditions, the fatigue life of pressure vessels becomes dispersed; thus, fatigue reliability methods are found to be better for data processing [45,46]. To further verify the new method proposed in this paper, the nominal stress method commonly used in engineering was used for comparison. The mathematical definition of the nominal stress method is given in Equation (20):

(20) $$\sigma_{n}=\frac{\sigma_{a}}{K_{T}}\varepsilon \beta C_{L}$$

where *σ_n_* is the stress of the mechanical structural part in the structural S-N curve, σ_a_ is the stress of material in the S-N curve, *K_T_* is the stress concentration factor, *ε* is the size factor, *β* is the surface processing coefficient, and *C_L_* is the loading coefficient. 

In this paper, the value of the size factor, ε, is 0.9, because the specimens are taken from the same nickel-chromium alloy material, so the value of the surface processing coefficient, β, is 1. The loading method of the fracturing pump is the same as in the case of the fatigue test, so the value of the loading coefficient is 1. In addition, based on Equations (18) and (20) and the S-N curve, the fatigue life prediction equation can be expressed as:

(21) $${\left(4.97\sigma_{n}\right)}^{8.1225}\cdot N=6.24\times 10^{26}$$

**Figure 18:**
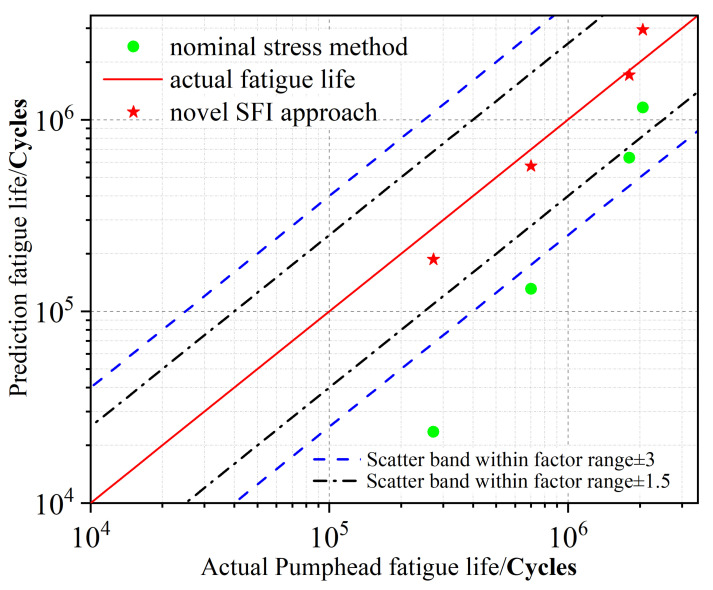
Pumphead fatigue life prediction accuracies of NSM and the novel SFI approach.

Therefore, in 65, 70, 80, and 105 MPa working conditions, combined with the S-N curves, fatigue life is calculated by the SFI approach and NSM, respectively. The fatigue life values of the pumphead under different conditions are displayed in Figure 18. It is evident that the novel SFI approach had the narrowest error band, indicating its higher fatigue life prediction accuracy for the pumphead.

## 5. Conclusions

Based on the theories of the critical distance method and the SFI approach, criteria for defining the fatigue damage failure region of the SFI approach were revisited. The proposed novel SFI approach manifested high fatigue life prediction accuracy for notch specimens and pumpheads. The main findings of this work are summarized below:

(1) Based on numerical simulation results and the concept of the critical volume method, an improved weight function accounting for the fatigue damage failure geometries of cardioid and crescent areas was proposed to improve fatigue life prediction accuracy.

(2) Based on the concept of the “invisible boundary”, the effective critical distance for crescent and cardioid areas of the fatigue failure region was revised and simplified. The novel SFI approach was modified considering the effect of grain size.

(3) To verify the fatigue life prediction effectiveness of the novel SFI approaches for notch specimens, fatigue tests were conducted on three different notched bar specimens. The novel SFI approach II had higher fatigue life prediction accuracy.

(4) In comparison with the SFI approach II, the SFI approach I was more suitable for fatigue life prediction of sharply notched specimens.

## Nomenclatures



$\sigma_{\max}$

Maximum stress
*k_t_*
Stress concentration factor

$\sigma_{FI}$

Stress-field intensity
*k_f_*
Fatigue notch factor

r

Distance to the peak stresspoint

$\bar{\chi }$

Stress gradient

$f(\sigma_{ij})$

Equivalent stress function

χ

Relative stress gradient

$\varphi (\vec{r})$

Weight function

$\sigma_{0}$

Nominal stress

φ(r,θ)

Weight function with distance *r* and deviation angle θ

$\varepsilon_{a}$

Strain amplitude

$\sigma_{a}$

Stress amplitude
*R*
 Field diameter σ_n_The load of component weakest regionσ_U_Ultimate stressσ_b_Ultimate tensile strengthσ_mean_Effective stress

$\Delta K_{th}$

Range of threshold value for fatigue crack propagation

$\Delta \sigma_{-1}$

Range of the fully reverse axial fatigue limit

$\sigma_{mean}$

Equivalent stress of TCD

$\sigma_{1}$

The maximum principal stressS_Y_Yield stress
*G*
Grain size function

ξ(Ω)

Obstructive effect

θ

Angular coordinates in Cartesian coordinatesσ*_yy_*(*r*)Fatigue crack-opening stress

$L_{0}$

Critical distance νPoisson’s ratio


**Abbreviations**



SFIStress-field intensityTCDTheory of critical distance methodLFEMLinear elastic fracture mechanicsEPFMElastic–plastic fracture mechanicsNSMNominal stress methodLSSMLocal stress–strain method

## Figures and Tables

**Table 1:** Empirical formulas for cyclic plastic zone size calculation.

Reference	Formula
Nicholls and Martin [27]	${r^{\prime }}_{y}=a\left(\frac{\sigma_{app}^{2}}{\sigma_{y}^{2}-\sigma_{app}^{2}}\right)$
Bathias and Pelloux [31]	${r^{\prime }}_{y}=0.1{\left(\frac{\Delta K}{\sigma_{y}}\right)}^{2}$
Pineau and Pelloux [28]	${r^{\prime }}_{y}=0.053{\left(\frac{\Delta K}{\sigma_{y}}\right)}^{2}$
Saxena and Antolovich [29]	${r^{\prime }}_{y}=\alpha {\left(\frac{\Delta K}{\sigma_{y}}\right)}^{2+s}$
Park et al. [30]	${r^{\prime }}_{y}=\frac{\pi }{144}{\left(\frac{\Delta K}{\sigma_{y}}\right)}^{2}$
Chapetti et al. [21]	${r^{\prime }}_{y}=\frac{1}{12\pi }{\left(\frac{\Delta K}{\sigma_{y}}\right)}^{2}$
Edmunds and Willis [33]	${r^{\prime }}_{y}=\frac{1}{24\pi }{\left(\frac{\Delta K}{\sigma_{y}}\right)}^{2}$

σ_app_ represents the applied stress, and ⍺ and s denote correlation and variation coefficients, respectively.

**Table 2:** Fatigue damage regions of different approaches.

Approach	Authors	Fatigue Damage Region	Influence Factors
Neuber’s and Peterson’s empirical formula	Neuber and Peterson [40,41]	Material constants, *a*_1_, *a*_2_	*a*_1_, *a*_2_ depend on fracture strength σ_b_
SED approach	Lazzarin and Berto [42]	Radius of critical volume (area), *R_0_*	*R_0_* depends on the threshold value of fatigue crack propagation, fatigue limit, Poisson’s ratio, and notch shape
SFI approach	Yao [41]	$\mathrm{Radius}\;\mathrm{of}\;\mathrm{the}\;\mathrm{fatigue}\;\mathrm{damage}\;\mathrm{field},\;\vec{r}$	$\vec{r}$ depends on grain size
Modified SFI approach	Qylafku [22]	Radius of the fatigue damage field, *r*	*r* depends on metallic-plastic deformation
TCD and M-TCD	Taylor and Susmel [19]	Intrinsic crack length, *l*_0_	*l*_0_ depends on the threshold value of fatigue crack propagation, fatigue limit, and stress ratio
Advanced volumetric method	Adib-Ramezani [10]	Effective distance, *X_eff_*	*X_eff_* depends on stress distributions at notch roots

**Table 3:** Static mechanical properties of nickel-chromium alloy.

Material	Serial Number	Young’s Modulus	Poisson’s Ratio	Yield Stress (s_Y_)	Ultimate Tensile Strength (*σ*_b_)
40Cr Ni_2_MoV	1	204 GPa	0.3	980 MPa	1060 MPa
40Cr Ni_2_MoV	2	204 GPa	0.3	980 + 3 MPa	1060 + 3 MPa
40Cr Ni_2_MoV	3	204 GPa	0.3	980 − 2 MPa	1060 − 3 MPa

**Table 4:** Empirical data of different fatigue test groups for the pumphead material.

Material	Stress (*S*_max_, MPa)	*N_f_* (Life)					$\mathrm{Median}\;(\bar{x})$
40CrNi_2_MoV	Level/count	1	2	3	4	5	——
	756.3	1037	1055	980	957	1123	1030.4
	716.5	5037	4958	5968	7853	6842	6131.6
	636.9	35,600	25,500	20,360	31,186	32,100	28,949.2
	517.5	110,200	175,100	125,900	140,500	152,900	140,920
	437.8	403,100	285,600	605,800	367,500	702,600	472,920
	398.1	321,580	715,600	456,800	555,200	985,600	606,956
	318.4	5,569,800	6,771,100		7,865,200		6.735 × 10^6^

**Table 5:** Cyclic loading parameters and results of fatigue test for 40CrNi_2_MoV notched specimens.

*Specimen No.*	*kt*	*F (kN)*	*Frequency (Hz)*	*Nf (Cycles)*	*Rσ*	*Median Life*
1-1	2	20 kN	10.0	62,724	−1	65,215
1-2	2	20 kN	10.0	68,563	−1	
1-3	2	20 kN	10.0	64,359	−1	
2-1	2	19 kN	10.0	122,133	−1	134,011
2-2	2	19 kN	10.0	134,572	−1	
2-3	2	19 kN	10.0	145,328	−1	
3-1	2	17 kN	10.0	363,235	−1	390,452
3-2	2	17 kN	10.0	384,527	−1	
3-3	2	17 kN	10.0	423,596	−1	
4-1	3	10 kN	10.0	6680	−1	6949
4-2	3	10 kN	10.0	6899	−1	
4-3	3	10 kN	10.0	7268	−1	
5-1	3	7 kN	10.0	85,362	−1	82,295
5-2	3	7 kN	10.0	81,237	−1	
5-3	3	7 kN	10.0	80,198	−1	
6-1	3	5.5 kN	10.0	638,340	−1	576,630
6-2	3	5.5 kN	10.0	534,568	−1	
6-3	3	5.5 kN	10.0	556,982	−1	
7-1	5	8 kN	10.0	36,024	−1	37,226
7-2	5	8 kN	10.0	45,396	−1	
7-3	5	8 kN	10.0	30,258	−1	
8-1	5	7.5 kN	10.0	104,417	−1	109,131
8-2	5	7.5 kN	10.0	109,853	−1	
8-3	5	7.5 kN	10.0	110,123	−1	
9-1	5	6 kN	10.0	190,823	−1	192,826
9-2	5	6 kN	10.0	198,095	−1	
9-3	5	6 kN	10.0	189,562	−1	

**Table 6:** Theoretical results of fatigue life for three notched specimens, calculated by four approaches.

Stress Concentration Factor	F/kN	Median Life	Novel SFI Approach I		Novel SFI Approach II		Traditional SFI Approach		Traditional TCD (LM)	
			σ_FI_/ MPa	*N_f_*/ Cycles	σ_FI_/ MPa	*N_f_*/ Cycles	σ_FI_/ MPa	*N_f_*/ Cycles	σ_mean_/ MPa	*N_f_*/ Cycles
kt = 2	20 kN	65,215	502.21	71,072	510.68	63,525	558.3	30,468	572.3	24,911
	19 kN	134,011	455.36	157,695	466.16	132,202	509.5	64,033	529.5	46,846
	17 kN	390,452	407.71	397,452	406.18	404,984	453.2	166,555	482.6	100,499
kt = 3	10 kN	6949	725.55	3649	706.44	4425	762.3	2428	809.2	1497
	7 kN	82,295	508.94	66,667	495.75	80,965	561.3	29,164	592.4	18,966
	5.5 kN	576,630	398.06	476,335	389.29	573,213	452.1	157,535	473.8	117,128
kt = 5	8 kN	37,226	540.68	39,948	521.21	53,424	596.7	17,747	611.5	14,545
	7.5 kN	109,131	478.02	107,568	469.18	125,477	530.4	46,194	562.9	28,506
	6 kN	192,826	425.79	279,319	416.39	332,268	472.9	117,319	498.6	77,084

## Data Availability

All data are presented in the article.
